# 

**Published:** 1874-01

**Authors:** 


### INDEX, VOL. XXVIII.

A System of Dental Surgery . . . . . . 50
American Academy of Dental Science . . 314, 48
Address . . . . . . . . . . 64
A new Method of Pivoting . . . . . . 68
Automatic Saliva Ejector . . . . . . . . 93
Associated effort . . . . . . . . 114
American Dental Society of Europe . . 117, 437
A Biographical Sketch of Dr. John Allen . . 141
A Bad Case . . . . . . . . . . 167
A Hint on Giving Iodide of Potassium . . . 168
A Test for Carbolic Acid . . . . . . . 178
A Letter . . . . . . . . . . 178
Abnormal Mucous Membrane . . . . . . 185
Address to the Graduates of the Ohio Dental College 202
Associations . . . . . . . . . 223
Annual Address of the Michigan Dental Association . 272
Attention . . . . . . . . . . 309
American Dental Association . 310, 389, 400, 420
American Dental Convention . . . 312, 402
A new style of Pivot Teeth . . . . . 361
An advance step for Celluloid . . . . 403
A new Dental Engine . . . . . . . 403
Annual Meeting Minnesota Dental Association . 433
Address delivered before the Academy of Dental Science 466

A Law to Regulate the Practice of Dentistry in the State of New Jersey . . . . . . . 485
A Manual of Dental Mechanics . . . . . 488
Anæsthetics—Three Deaths from Chloroform and Ether 500
Filling Frail Teeth with Gold . . . . . . 502
Attention . . . . . . . . 490
A new Hand Piece . . . . . . 491

Book Reviews . . . . . . . 39, 88
Bibliographical . . . . . . . 184
Barnum's Coffer-Dam . . . . . . 301
Bind your Dental Journals . . . . . 382
Baxter's Automatic Mallet . . . . . 484
Burs and Excavators . . . . . . 492

Contour Fillings . . . . . . . 53, 104
Conservative Treatment of the Dental Pulp . . 317
Cheap Polishing Wheels and Cones . . . 336
Commencement . . . . . . . 96
Correspondence . . . . . . . . 477

Diagnosis . . . . . . . . . 9, 71
Discussions . . . . . . . . . 126, 150
Dental Engines . . . . . . . 270
Death of Dr. Mendenhall . . . . . 396
Death from Chloral . . . . . . 316
Dental Ethics . . . . . . . 331
Defects in the Palatine Organ . . . . . 384
Dental Floss Holders . . . . . . 404
Diversity of Experience in Dental Practice . . 440
Dental Convention . . . . . . . 490
Dentistry in the Exposition . . . . . 510
Dental Vulcanite Controversy . . . . . . 526

Eastern Ohio Dental Association . . . . . 162
Education . . . . . . . . 285
Exposed Nerves . . . . . . . 302
Experiment in Liquefying Gases . . . . . 520

Fatal Poisoning from Carbolic Acid . . . 18
Found at Last . . . . . . . . . 47
Free Mercury in Amalgam Fillings . . . 238
For Sensitive Dentine . . . . . . . 530
Fungoid . . . . . . . . . 531
Failures . . . . . . . . . 525

Gold Caps . . . . . . . . . 92
Good Potable Water . . . . . . 175

How much of the Success of Filling Teeth is due to Manipulation . . . . . . . 33
How to deprive Iodine of its Stain . . . . . 134
History of Dentistry . . . . . . 139
Hernia of the Dental Pulp . . . . . . 198
Have Smooth-faced pluggers any advantage over Serrated? . . . . . . . . 458

Illinois State Dental Society . . . . . 226, 269
Improvements in Working Rubber . . . . 521
Improvements in Operative Dentistry. What is demanded of us, and what we should expect . 229
Important to Medical Students . . . . . 402
Introductory Address before the Class in the Ohio Dental College . . . . . . . . 493
Is it a good practice to leave the Dentinal Tubuli open? 283

Journalistic . . . . . . . . . 95

Kentucky State Dental Society . . . . . 315

Lime Phosphates . . . . . . . . 108

Law and Medicine . . . . . . . . 166

Letter from China . . . . . . . 214

Lacto Phosphate of Lime . . . . . . 480

Merrimack Valley Dental Association . . . . 483

Minutes of the Ohio State Dental Society . . 75

Michigan Dental Association . . . . . . 513

Mississippi Valley Dental Society . . . 183, 95
Mortality of various Cities . . . . . . 167
Mad River Valley Dental Society . . 388, 401, 176

Neuralgia caused by Granules of Osteo-Dentine in Pulp Cavaties of Teeth . . . . . . 39
Nickel Plating . . . . . . . . 160
Northern Ohio Dental Association . . 271, 340
New Instruments . . . . . . . 319
Notice . . . . . . . . . 447
Necrosis—A strange Case . . . . . 456

Ohio State Dental Society . . . . 4 488
Oatmeal, Bone and Muscle . . . . . 73
Ohio Dental College Association . . . . 96
Opening of the Pulp Cavity and preparation of the Roots for filling . . . . . . 67
Ohio Dental College Commencement . . . 140
Oriental Wisdom . . . . . . . 177
Obituary . . . . . . . 185, 359, 404
Ohio College of Dental Surgery . . . . 314
On the relation of Dentist and Patient . . . 373
Palmer's Instruments . . . . . . 52
Ohio Dental College Laboratory . . . . 528

Proceedings of Mad River Valley Dental Society . 518
Personal . . . . . . . . . 52
Professional Pride . . . . . . . 59
Popular Instruction . . . . . . 94
Production of Iodine in France . . . . . 174
Principal Cause of Constitutional Derangement in first Dentition . . . . . . . . 295
Proceedings of Tennessee Dental Association . 345
Progression and Retrograde Metamorphosis of Bones and Teeth . . . . . . . 405

Radical observations about People's Teeth . . 16
Retiring Address . . . . . . . 33

Revelations of the Microscope . . . . . 196
Reply on behalf of the Class of 1874 . . . 211
Revival in Dental Journalism . . . . . . 217
Rubber Dam . . . . . . . . . 268
Rubber Litigation . . . . . . . . 271
Resolutions of Respect . . . . . . . 272
Report on Dental Education . . . . . 325
Remarks on the Advance in Practice and Use of Mechanical Appliances . . . . . . . 504

Sensitive Dentine and its Remedy . . . 59, 148
Screws . . . . . . . . . . 137
Selected Abstracts . . . . . . . . 186
Southern Dental Association . . . . . . 311
South Carolina Dental Association . . . 433
Synopsis of the Discussion of the American Dental Society in Europe . . . . . . . 472

The History of Dentistry . . . . . . 492
The Deciduous Teeth and their Treatment—Causes of Irregularity of the Permanent Teeth . . 19
The Pathology of the Teeth . . . . . . 41
The Pulse of various Animals . . . . . 15
Success , . . . . . . . . . 523
The Physician's Visiting List . . . . . 532
The Diarrhœa of Teething Children . . . 72
The Process of Embalming . . . . . . 74
The Microscope . . . . . . . . 112
The proper use of Cholagogues . . . . 134
The Morrison Engine Infringement . . . 138
The Magic Latern an aid to Instruction in Chemical and Physical Lectures . . . . . . . 172
The Health of the Dentist . . . . . . 180
To our Contributors . . . . . . . 219
To the Profession . . . . . . . . 220
The Enamel . . . . . . . . . 241
The District Dental Society of the 7th Judicial District of the State of New York . . . . 336

The Sixth Annual Meeting of the Dental Society of the State of New York . . . . . . 338
The Education of the Dentist . . . . . 369
The Dental Student . . . . . . . 377
The Dental Colleges . . . . . . . 441
Treatment of Diseased Gums . . . . . . 443
Twelfth Annual Meeting of the Connecticut Valley Dental Society . . . . . . . . 448

What does Experience teach to be the best Material for filling Teeth . . . . . . . 449
Which would be Right . . . . . . 289
Why Not ? . . . . . . . . . 415
Vulcanite Litigation . . . . . . 90, 445

### PERSONAL.

Within the last two months' Dr. Corydon Palmer has removed from Warren, Ohio, to New York City, No. 102 West 38th St., where with his son, Dr. Delos Palmer, he has opened an office, and is engaged in practice.

The Dr. is more nearly in his element than he has been for a long time, We trust this move will not prevent him from responding to calls that may be properly made for his services as a clinical teacher, in any of our professional societies throughout the country to which he may be called. In a practical way, Dr, Palmer is one of our most eminent teachers, and the societies should avail themselves of his assistance whenever at all practicable. We hope he will realize success in his new field beyond his highest expectations.

### CONTENTS:

### COMMUNICATIONS.

Diagnosis .................................................. 9

Radical Observations about People's Teeth, No II ...... 16

The Deciduous Teeth and their Treatment—Causes of Irregularity of the Permanent Teeth, etc ............. 19

How Much of the Success of Filling Teeth is Due to the Manipulation ........................................ 33

Retiring Address ........................................ 36

Neuralgia Caused by Granules of Osteo Dentine in Pulp Cavities of Teeth ........................................ 39

Book Reviews:

The Pathology of the Teeth .......... ............ 41

A System of Dental Surgery ........................ 43

### PROCEEDINGS OF SOCIETIES.

American Academy of Dental Science ................. 45

### MISCELLANEOUS

The Pulse of Various Animals ........................ 15
Fatal Poisoning from Carbolic Acid ................... 18

### EDITORIAL.

Found at Last ............................................. 47

Ohio State Dental Society ................................. 49

United States Circuit Court—The Dental Rubber Cases . 51

Palmer's Instruments ...................................... 52

Personal ................................................... 52

---

### THE DENTAL REGISTER,

### OF THE WEST.

### A Monthly Magazine, Devoted Entirely to the Interest of the DENTAL PROFESSION.

**Terms Reduced to $2.50 per Year. Single Nos. 25 Cents.**

**Edited by PROF. J. TAFT,**

And Published by SPENCER & MOORE, of the Ohio Dental Depot

The volume commences in January and closes in December.

The Register being issued monthly is always fresh, therefore indispensable to the practitioner who desires to keep posted up with the new inventions and improvements which are daily developed. Neither expense nor effort will be spared to give value and variety to its contents. Articles will be illustrated with well executed engravings whenever they will add to the interest or assist in explanation.

Its extensive circulation and frequency of issue makes it one of the BEST DENTAL ADVERTISING MEDIUMS in the United States.

All subscriptions must begin with January or July.

**RATES OF ADVERTISING:**

| | 1 mo. | 3 mo. | 6 mo. | 1 year. | | 1 year. | |
|---|---|---|---|---|---|---|---|
| 1 page ......... | $12 00 | $25 00 | $40 00 | $75 00 | Inserted pages must be plain | 1st page. | $50 00 |
| ½ page ......... | 8 00 | 15 00 | 25 00 | 40 00 | paper & black | 2d page. | 37 50 |
| ¼ page ......... | 5 00 | 10 00 | 15 00 | 25 00 | ink. | 3d page. | 33 33 |
| | | | | | | 4th page. | 25 00 |

Local or special notices, five lines or less, will be inserted for $3 each insertion; over five lines, 50 cents per line.

All advertising bills payable in advance, or at furthest, after the issue of the first number containing the advertisement. All transient advertisements to be paid for in advance.

☞CONTRIBUTIONS SOLICITED.

Exclusively Editorial Communications should be addressed to

**DR. J. TAFT, EDITOR.**

The latest number of the Register may be ordered with or without other goods at any time, and all letters should be addressed to

**SPENCER & MOORE. PUBLISHERS,**

Cincinnati, Ohio.

